# COVID-19’s impact on care practice for alpha-1-antitrypsin deficiency patients

**DOI:** 10.1186/s12913-023-09094-3

**Published:** 2023-01-30

**Authors:** Myriam Calle Rubio, José Luis López-Campos, Marc Miravitlles, Francisco Javier Michel de la Rosa, José María Hernández Pérez, Carmen Montero Martínez, José Bruno Montoro Ronsano, Francisco Casas Maldonado, Juan Luis Rodríguez Hermosa, Eva María Tabernero Huguet, José Manuel Martínez Sesmero, Carlos Martínez Rivera, Francisco Javier Callejas González, María Torres Durán

**Affiliations:** 1 Pulmonology Department, Health Research Institute of the Hospital Clínico San Carlos (IdISSC), San Carlos Clinical Hospital, Madrid, Spain; 2 Department of Medicine, School of Medicine, Universidad Complutense de Madrid, Madrid, Spain; 3 Medical-Surgical Unit for Respiratory Diseases, Biomedicine Institute of Sevilla (IBiS), Virgen del Rocío University Hospital, Seville, Spain; 4 Pulmonology Department, Research Institute of Vall d’Hebron (VHIR), Vall d’Hebron University Hospital, Barcelona, Spain; 5 Network of Centers for Biomedical Research On Respiratory Diseases (CIBERES), Carlos III Health Institute, Madrid, Spain; 6 Pulmonology Department, Donostia University Hospital, San Sebastián, Spain; 7 Pulmonology Department, Nuestra Señora de Candelaria University Hospital, Santa Cruz de Tenerife, Spain; 8 Pulmonology Department, A Coruña University Hospital, A Coruña, Spain; 9 Department of Pharmacy, Vall d’Hebron University Hospital, Barcelona, Spain; 10 Pulmonology Department, San Cecilio Clinical University Hospital, Granada, Spain; 11 School of Health Science, University of Granada, Granada, Spain; 12 Pulmonology Department, Cruces University Hospital, Barakaldo, Spain; 13 Department of Pharmacy, San Carlos Clinical Hospital, Madrid, Spain; 14 Pulmonology Department, Research Institute of Germans Trias I Pujol (IGTiP), Germans Trias I Pujol University Hospital, Barcelona, Spain; 15 Pulmonology Department, Albacete University Hospital Complex, Albacete, Spain; 16 Pulmonology Department, Health Research Institute of Galicia Sur (IISGS), Álvaro Cunqueiro Hospital, Estrada Clara Campoamor, 342. 36312 Vigo, Spain

**Keywords:** COVID-19, Alpha-1-antitrypsin deficiency, Recommendations, Patient management, Healthcare system, Rare disease, Diagnostic, Treatment, Follow-up, AATD

## Abstract

**Background:**

Patients with alpha-1 antitrypsin deficiency (AATD), commonly categorized as a rare disease, have been affected by the changes in healthcare management brought about by COVID-19. This study’s aim was to identify the changes that have taken place in AATD patient care as a result of the COVID-19 pandemic in Spain and to propose experts’ recommendations aimed at ensuring humanized and quality care for people with AATD in the post-pandemic situation.

**Methods:**

A qualitative descriptive case study with a holistic single-case design was conducted, using focus groups with experts in AATD clinical management, including 15 health professionals with ties to the Spanish health system (12 pneumologists and 2 hospital pharmacists from 11 different hospitals in Spain) and 1 patient representative.

**Results:**

COVID-19 has had a major impact on numerous aspects of AATD clinical patient management in Spain, including diagnostic, treatment, and follow-up phases. The experts concluded that there is a need to strengthen coordination between Primary Care and Hospital Care and improve the coordination processes across all the organizations and actors involved in the healthcare system. Regarding telemedicine and telecare, experts have concluded that it is necessary to promote this methodology and to develop protocols and training programs. Experts have recommended developing personalized and precision medicine, and patient participation in decision-making, promoting self-care and patient autonomy to optimize their healthcare and improve their quality of life. The possibility of monitoring and treating AATD patients from home has also been proposed by experts. Another result of the study was the recommendation of the need to ensure that plasma donations are made on a regular basis by a sufficient number of healthy individuals.

**Conclusion:**

The study advances knowledge by highlighting the challenges faced by health professionals and changes in AATD patient management in the context of the COVID-19 pandemic. It also proposes experts’ recommendations aimed at ensuring humanized and quality care for people with AATD in the post-pandemic situation. This work could serve as a reference study for physicians on their daily clinical practice with AATD patients and may also provide guidance on the changes to be put in place for the post-pandemic situation.

## Background

The ongoing coronavirus disease 2019 (COVID-19) pandemic has disrupted every aspect of our lives. The need to provide high-level care to an enormous number of patients with the COVID-19 infection during this pandemic, and the restrictions and lockdowns imposed by governments, has impacted resources and restricted the routine care of all non-COVID-19 conditions [1]. Since the beginning of the pandemic, people living with chronic disorders have not received the necessary attention that they deserve, this being even more serious in the case of patients with rare diseases [2–4]. Patients with alpha-1 antitrypsin deficiency (AATD), commonly categorized as a rare disease, have been one of the groups affected by the changes in healthcare management brought about by COVID-19. AATD is one of the most common genetic disorders leading to a wide spectrum of clinical manifestations, ranging from no symptoms to a progressively debilitating systemic disease, most commonly affecting lung and liver [5]. Adult AATD patients tend to present the usual symptoms of chronic obstructive pulmonary disease (COPD), but with an earlier onset: cough, expectoration, dyspnea and frequent exacerbations, although the main symptom is progressive dyspnea [6]. AATD’s estimated prevalence is between 1:2500 and 1:5000 according to studies from the United States and Europe [7, 8], but it is necessary to emphasize that it's estimated that a large number of patients are undiagnosed [9], and also that there’s a long delay between symptom onset and AATD diagnosis [10].

Recent publications have linked AATD to COVID-19. It’s been suggested that AAT may act as a protective factor against the disease for several reasons, such as its inhibition of the TMPRSS2 and ADAM17 proteases, which are key to the virus’s pathophysiology [11, 12]. AAT’s role in SARS CoV-2 cell infection has been studied, and clinical trials are underway to examine AAT’s potential usefulness as a treatment for COVID 19 [13]. On the other hand, the frequently associated comorbidities in AATD patients, including a higher prevalence of hypertension, chronic kidney disease, COPD, and diabetes, could predispose patients to severe COVID-19 and a poor prognosis [14, 15]. Consequently, AATD patients would be a vulnerable group for infection with the virus [16]. Change in health system routines due the COVID-19 pandemic and the possible relationship between AATD and COVID-19 infection means it’s necessary to study the effects on AATD patient management. Unfortunately, there’s a knowledge gap in this regard, as no such studies have been carried out to date.

Therefore, this study’s aim was to identify the changes that have taken place in AATD patient care as a result of the COVID-19 pandemic in Spain, and to propose experts’ recommendations aimed at ensuring humanized and quality care for people with AATD in the post-pandemic situation.

## Methods

For this study, guidelines for conducting qualitative studies established by the Consolidated Criteria for Reporting Qualitative Research (COREQ) were followed [17].

### Design

A qualitative descriptive case study with a holistic single-case design was conducted [18], using focus groups (FGs) with experts in AATD clinical management. Qualitative methods are suitable for recognizing the beliefs, values, and motivations that underlie individual actions and behaviors [19]. A case study is a type of research design that studies a specific phenomenon in a real-life setting, and it can be used to identify and describe experiences regarding care and diseases [18]. A case study may consist of several units, which together describe a phenomenon in a complete way. These units may be different participants or experts who are linked by the phenomenon under study [18]. In this project, the phenomenon under examination is COVID-19’s impact on AATD patient management. Two focus groups were conducted, the first was held to evaluate the changes brought about by the pandemic situation, as well as the new needs and recommendations aimed at ensuring humanized and quality care for people with AATD in the current and post-pandemic situation. In the second focus group, the work carried out during the first workshop was shared with a wider number of experts with the aim of gathering additional contributions and reaching a consensus regarding the pandemic’s impact on the AATD care process and the unmet needs that have been revealed, as well as the recommendations issued in this regard for the post-pandemic situation. Finally, the insights obtained from both focus groups were compiled and summarized differentiating between impacts (positive and negative), unmet needs and recommendations.

The study duration was from March 1^st^, 2021, to August 1^st^, 2021.

### Participants

The inclusion criteria consisted of 14 professionals who have direct and extensive experience in the clinical management of AATD patients’ (12 pneumologists and 2 hospital pharmacists from 11 different hospitals in Spain) and a patient’s representative. Participants were divided into two groups: the core group was made up of 3 experts (2 pneumologists and 1 hospital pharmacist) who acted as study coordinators and participated in both FGs. The rest of the participants took part in the second FG. In addition, the patients’ perspective was considered. For this purpose, an in-depth- interview with the President of the Spanish Patients’ Association (Alfa 1 Spain) who is a patient himself. All the experts reviewed and validated the documentation generated.

### Data collection

In order to examine different perspectives within the same group, FGs were held to gain an understanding of the problems faced by the group and to aid identification of values and norms [20]. This method of data collection is consistent with the case study’s design [18]. The first FG was held on May 4^th^,2021, while the second FG was conducted on May 25^th^,2021.

## Results

### The COVID-19 pandemic’s impact on AATD Diagnosis management

Experts stated both positive and negative effects of the COVID-19 pandemic on the AATD diagnosis stage (Table 1).

**Table 1:** Positive vs negative effects of COVID-19 pandemic on AATD diagnostic stage

**Positive efects of COVID-19 on AATD diagnosis**
Development of telephone consultations and other remote assistance channels as a method for professionals to assess patients
**Negative efects of COVID-19 on AATD diagnosis**
Delays and cancellations of diagnostic tests
Delays on the initial visits to the pneumologists, which are of great importance for making a correct diagnosis
Lack of protocols, tools and resources for remote assistance
Greater difficulty for effective communication and coordination between primary care and hospital care

### The COVID-19 pandemic’s impact on AATD treatment management

In this phase of treatment management, the changes in protocols brought about by the COVID-19 pandemic have shown several positive aspects, as expressed by experts. However, there has been also an important negative impact which have resulted in new issues or exacerbated pre-existing ones. (Table 2).

**Table 2:** Positive vs negative effects of COVID-19 pandemic on AATD treatment management

**Positive efects of COVID-19 on AATD treatment**
Breakdown of existing administrative barriers, thus facilitating home delivery of medication and home-based therapy
Positive change of attitude and convincement for both patients and healthcare professionals with respect to auto-administration of replacement treatment
Lower incidence of influenza and other respiratory viruses because of the lockdown and COVID-19-related measures
Increase in smoking cessation
**Negative efects of COVID-19 on AATD treatment**
Decline in blood donations due to the security measures adopted by hospitals, redistribution of resources, high pressure of critical patient care and reluctance to donate blood, among other reasons
Delays on treatment due to healthcare services saturation, leading some patients to follow a suboptimal treatment regimen
Increased difficulties in instructing patients to change inhalers and escalate treatments
Increased difficulty in assessing lung transplantations
Changes in lifestyle related to lockdown measures which have resulted in problems for patients keeping a healthy lifestyle and a good diet. These changes have also impacted negatively patients’ mental health

### The COVID-19 pandemic’s impact on AATD Follow-up management

The development of new tools for remote assistance have been an important advancement during COVID-19 for AATD patients in the different stages of the care process, especially for follow-up. However, experts identified also some negative effects of the pandemic in patient care during this stage (Table 3).

**Table 3:** Positive vs negative effects of COVID-19 pandemic on AATD follow-up

**Positive effects of COVID-19 on AATD follow-up**
Promotion and positive uptake of remote assistance for patient follow-up, accompanied with the development of new tools for maintaining the continuity of care
**Negative effects of COVID-19 on AATD follow-up**
Delays and cancellations of follow-up visits due to healthcare services’ saturation
Reduced number of complementary tests for patients’ follow-up
Lack of protocols, tools and resources for remote assistance, which have led to a sense of uncertainty in patients

### The COVID-19 pandemic’s all-round impact on AATD management

Expert identified certain across-the-board aspects on AATD management that have been affected by COVID-19 pandemic, some of them in a negative way whereas others are positive for AATD management (Table 4).

**Table 4:** Cross-board impact of COVID-19 pandemic on AATD management

**Positive cross-board effects**
Organization of online seminars to reinforce patient’s information and reduce their uncertainty
Use of different media and social networks to give best public health advice and recommendations to patients who need it
Better communication between different care levels
Increased involvement and participation of patients’ associations in the dissemination of information, providing support to patients and strengthening psychosocial monitoring
**Negative cross-board effects**
Mistrust on going to the health center for fear of infection
Problems in differentiating between patients with respiratory conditions and suspected COVID patients, thus being admitted incorrectly to the special COVID circuit, and consequently making it difficult to access appropriate consultations
Professionals’ feeling of loneliness during the pandemic, highlighting the need to establish coordinated criteria among them for decision-making, in line with legal and ethical principles
Emotional tension and stress among professionals, the lack of official documents and criteria for managing the pandemic, and treatment continuity for patients with chronic diseases

### Needs identified in AATD clinical management due to the COVID-19 pandemic

As a consequence of this health crisis caused by the COVID-19 pandemic, a series of challenges have been identified by the experts regarding the AATD care process (Table 5).

**Table 5:** Identified needs on the clinical management of AATD due to the COVID-19 pandemic

1. Strengthening Primary Care and Hospital Care: the overflow of health services has caused problems such as delays in diagnosis and/or treatment or problems in follow-up, especially in rare diseases
2. To promote self-care and patient autonomy to optimize their healthcare and improve their quality of life
3. To progress in the coordination and communication between the healthcare systems of the different Autonomous Communities, between healthcare levels, and between the different agents of the system (public administration, healthcare, industry, and patient associations)
4. Networking and collaboration between different specialties when forming care teams to improve the approach to pathologies such as AATD
5. Develop personalized and precision medicine as a tool for the identification and application of a personalized preventive, diagnostic and therapeutic approach to patients with AATD, as well as patient participation in decision-making
6. To ensure that plasma donations are made on a regular basis by a sufficient number of healthy individuals
7. Promote telemedicine and telecare to offer patients who so wish the possibility of complementing their face-to-face care with remote care
8. Encourage initiatives that promote a holistic conception of healthcare

### Recommendations for AATD management in the post-pandemic situation

After identifying COVID-19’s impact on AATD patients’ clinical management, the experts concluded with several recommendations for improving the post-pandemic situation (Table 6).

**Table 6:** Recommendations for the management of AATD in the post-pandemic situation

1. Reinforce Primary Care and Hospital Care health services, both in terms of staff and resources, with the aim of minimizing the cancellations and postponements observed during the health crisis, which delay diagnosis, treatment, and follow-up, especially for rare diseases such as AATD
2. Develop health education programs and information materials for patients, aimed at increasing their awareness of AATD to foster their autonomy: a. Achieve patient self-detection of alarm symptoms, exacerbations, etc b. Inform about the benefits of regular exercise and optimal nutrition for disease management to counteract the inactivity caused by the confinement c. Maintain awareness-raising messages on the risk of tobacco use, taking advantage of the increase of smoking cessation produced during confinement d. Inform about the harmful effects of alcohol in order to discourage its consumption e. Raise awareness of the advantages of maintaining in certain cases the measures taken against coronavirus to avoid infection by other respiratory viruses and to protect against harmful substances in the environment that may aggravate COPD
3. Ensure the possibility of self-administration of treatment to increase patient autonomy and self-care. This type of treatment has already demonstrated several positive points in other pathologies (e.g. in hereditary angioedema) and is supported by healthcare professionals at the European level
4. Ensure the availability of TAA replacement therapy from home and inform patients of this possibility. In this way, those patients who prefer this option will be able to opt for it to increase their quality of life, whilst reducing the burden of care on the health center
5. Develop and implement coordination and communication strategies between the different levels of care, between different specialties, as well as between the different agents in the system to improve the approach to the AATD care process
6. Promote the development of personalized and precision medicine in national and regional health strategies and plans. The pandemic has highlighted the relevance of personalized treatments and, in the case of AATDs, it is particularly important due to the variability of their natural history
7. Prescribe individualized physical activity programs, taking into account the capacities and needs of each patient, with the aim of subverting the bad habits acquired during the pandemic, as well as assessing the nutritional status of the patient individually (dietary, anthropometric, and hematological studies, etc.) for the early identification of those patients at a greater risk of malnutrition and establish the degree of nutritional support recommended
8. Promote the development of plasma donation campaigns in coordination with blood banks and raise public awareness of the importance of plasma donation for this type of treatment, such as AATD
9. Develop monitoring instruments that can be used from the patient's home and provide sufficient information for decision-making in the AATD care process (questionnaires in apps, spirometers incorporated into mobile devices, e.g.) to promote telemedicine
10. Develop training programs in telecare for healthcare workers. In this regard, it is recommended that resources for telecare be increased, so that they are accessible to patients as a complementary tool to face-to-face care
11. Promote psychosocial support, continuous and personalized communication with the patient, assisting patients from a holistic perspective, taking into account their clinical, social, and personal characteristics, their needs, etc

## Discussion

Although there are studies on COVID-19’s impact on COPD management as well as on rare diseases [2, 21], to the best of our knowledge this is the first study to analyze COVID-19’s impact on AATD patient management. Our findings revealed that COVID-19 has had a major impact on numerous aspects of clinical patient management in Spain, including the diagnostic, treatment and follow-up phases. In addition, the pandemic context has highlighted various needs in AATD patient care; experts have therefore drawn up a series of recommendations in order to meet these needs and address the impact caused by COVID-19.

An overall view of the results highlights some aspects repeated in several sections of the study. One of them is that COVID-19 has caused cancellation or postponement of a large number of medical procedures, impacting AATD patients’ diagnosis, treatment and follow-up phases. As a result of health service saturation due to the pandemic, chronically ill patients have had their hospital and primary care consultations cancelled [22]. In Spain, a survey estimated that 43% of respiratory patients have been unable to make face-to-face appointments with a specialist since the first months of 2020 [23], a fact that supports the perception shown by this study’s experts. Another study concluded that there was a reduction in the number of complementary healthcare tests conducted due to the COVID-19 pandemic in COPD [24], and rare metabolic patients [3]. Therefore, as described by the experts in their recommendations, it would be necessary to strengthen both primary and hospital care in order to prevent these cancellations and delays, which can cause great harm to health outcomes in AATD patients.

Another aspect highlighted in this study was telemedicine/telecare’s implementation and growth. On the one hand, this has had a positive impact, as it’s enabled awareness and implementation of new tools for the health system that didn’t used to exist. However, its implementation was forced without prior planning and this is linked to feelings of insecurity in patients, who expressed a mixed perception of this type of care [25]. Implementation of telehealth is a challenge for managing patients with chronic respiratory diseases, as shown in a recent study [26]. One of the ways to access these tools’ full potential would be to improve health professionals’ training in telehealth. In this respect, there are several studies that even point to the need to include telemedicine training in the medical school curriculum [27, 28].

Another issue that’s recurred throughout this study is the relevance of and need for patients to have an active role not only in decision-making but also in boosting their autonomy and self-care. According to experts, patient education both on self-diagnosis and self-treatment is necessary and beneficial, based on the experiences lived during the COVID-19 pandemic. Regarding this aspect, there’s a need to make home-care available for the AATD treatment. The aim should be to reduce the number of doses of replacement therapy, shorten administration times and offer the possibility of administering it at home in order to increase the quality of life of those patients who prefer not to go to the health center [5, 23]. In this regard, the possibility of monitoring patients from home by developing instruments that can be used from patients’ homes and provide information for decision-making in the AATD care process (e.g., questionnaires on apps, spirometers incorporated into mobile devices) was also proposed as a recommendation by experts. With the rise of handheld spirometers, at-home spirometry has become common for daily monitoring of the amount and/or speed of air that can be inhaled and exhaled [29]. This could be a functional tool used in AATD patients’ home monitoring, enabling health system resources to be freed up while improving patient follow-up.

Personalized medicine was another point that came up during the study on several occasions. In the field of AATD patient management, there’s currently increasing interest in personalized medicine, especially regarding ATT treatment, and including the use of extended dosing intervals and at-home treatment [30, 31]. In the near future, increased patient stratification will allow for enhanced application of personalized medicine and pro-active treatment regimens, resulting in reduced costs and improvement in quality of life [32]. Experts have therefore recommended the need to boost development of personalized medicine for AATD patient management.

Another aspect relating to treatment availability are blood transfusions, which during the pandemic have suffered numerous cancellations due to the decrease in the number of donations in Spain, as attested by the study carried out by García-Erce et al. [33]. The importance of this resource, not only for clinically managing AATD [5, 34], but also in other medical situations [35, 36], has prompted experts to emphasize this aspect in several sections of this study as well as in the recommendations.

Regarding the healthcare system, on several occasions during the study, experts expressed the need and recommendation to increase and improve the coordination processes across all the organizations and actors involved. In the end, networking and collaboration between different specialties should be encouraged when forming care or working groups in order to improve the approach to pathologies such as AATD. In this regard, it should be noted that the Spanish healthcare system is quite complex, due in part to its administrative structure, which sometimes complicates coordination between its different units [37–39]. It’s therefore necessary to implement coordination measures that help to overcome barriers to achieving more effective and better integrated patient management.

This study has several limitations. Firstly, the results probably cannot be directly extrapolated onto other health systems. Therefore, this study’s recommendations can be implemented directly in the Spanish context but might not be appropriate for other countries or regions. Clinicians with experience in other healthcare systems should assess whether each recommendation in this study can be transferred onto their clinical practice and adapted or discarded by those who don’t consider it appropriate. Following the same methods as described in this study will enable other researchers to apply the same approach in other contexts. Another possible limitation in the study is to do with the limited number of health professionals who took part in the FGs. Although we tried to make it representative and diverse, there’s always the possibility of bias, since not all health professionals involved in AATD in Spain were represented. Additionally, another possible limitation is the use of online platforms to conduct the FGs, due to the restrictions caused by the COVID-19 pandemic. This could lead to some communication bias, although this technology may also have advantages such as broadening participation [40]. Lastly, it’s important to highlight that, even though it is predictable that the experts’ recommendations could have a positive impact in the clinical care of these patients, they would need to be tested before concluding their utility in real clinical practice.

## Conclusions

The study advances knowledge by highlighting the challenges faced by health professionals and changes in AATD patient management in the context of the COVID-19 pandemic. Though many of the issues and problems identified were also present before the pandemic, they have been exacerbated and are of greater concern in the pandemic situation. The study also proposes experts’ recommendations aimed at ensuring humanized and quality care for people with AATD. It could therefore serve as a reference study for all those physicians who’ve had to suffer COVID-19’s impact on their daily clinical practice in AATD patient management, and may also provide guidance on the changes to be put in place for the post-pandemic situation.

## Data Availability

All data generated or analysed during this study are included in this published article.

## Abbreviations

AATD: Alpha-1 antitrypsin deficiency
COREQ: Consolidated criteria for reporting qualitative research
COVID-19: Coronavirus disease 2019
COPD: Chronic obstructive pulmonary disease
FGs: Focus groups
